# Sarracenia Purpurea as a Remedy for Small-Pox

**Published:** 1863-09

**Authors:** 


### SARRACENIA PURPUREA AS A REMEDY FOR SMALL-POX.

In a letter to *The Times* of Tuesday last, Surgeon-Major Logie, Royal Horse Guards, (Blue) stationed at Windsor, writes that "Some time ago, seeing a paper written by Assistant-Surgeon Miles," of the Royal Artillery, on the efficacy of the North American plant called the *Sarracenia purpurea*, or pitcher plant, in the treatment of small-pox among the Indians, my colleague (Mr. Agnis) and myself have given this remedy, which has been imported into this country by Mr. Miles to the house of Messrs, Savory & Moore, a fair trial; and I am happy to say the eleven cases in our hands have recovered under its peculiar influence. This remedy I consider a boon to the public, for this reason—it is so easily managed; any one can make a decoction or infusion of the root, like tea. An ounce of the root is sliced and infused in a quart of water, and allowed to simmer down to a pint; this is given in two tablespoonful doses every four hours, while the patient is well nourished with beef-tea and arrowroot. Four of the cases in my hospital have been severe confluent cases; they have throughout the disease all been perfectly sensible, have had excellent appetites, been free from pain, and have never felt weak. The effects of this medicine, which I have carefully watched, seemed to arrest the development of the pustules, killing, as it were, the virus from within, thereby changing the character of the disease and doing away with the cause of pitting, and thus avoiding the necessity of gutta-percha and india-rubber applications, or of opening the pustules. In my opinion, all anticipations of disfigurement from pitting may now be calmed, if this medicine is given from the commencement of the disease. Before leaving this subject, I may here caution the public that the useful part of the plant is its root, as recommended by Mr. Miles; and it can only be obtained from Messsrs. Savory & Moore, to whose house alone it has been imported. With the usual kindness of Dr. Gibson, the Director-General, I have been amply supplied with it for the use of my regiment. So much am I impressed with the efficacy of it in small-pox over the old mode of treatment that I hope to hear of it in every country gentleman's medicine-chest, and before long that we shall see no more faces, as described by Dickens, like the interior surfaces of sliced muffins."—*London Lancet.*

